# In-Depth Analysis of Chlorophyll Fluorescence Rise Kinetics Reveals Interference Effects of a Radiofrequency Electromagnetic Field (RF-EMF) on Plant Hormetic Responses to Drought Stress

**DOI:** 10.3390/ijms26157038

**Published:** 2025-07-22

**Authors:** Julian Keller, Uwe Geier, Nam Trung Tran

**Affiliations:** 1 Forschungsring e.V., 64295 Darmstadt, Germany; 2 Applied Plant Sciences, Department of Biology, Technical University Darmstadt, 64287 Darmstadt, Germany; tran@bio.tu-darmstadt.de

**Keywords:** radiofrequency electromagnetic fields, lettuce, photosynthesis, OJIP, fresh and dry matter, drought, machine learning, anomaly detection, plant stress

## Abstract

The proliferation of telecommunication devices in recent decades has resulted in a substantial increase in exposure risk to manmade radiofrequency electromagnetic fields (RF-EMFs) for both animals and plants. The physiological effects of these exposures remain to be fully elucidated. In this study, we measured and analyzed the chlorophyll fluorescence rise kinetics of lettuce plants in the presence of RF-EMFs and after a short drought treatment. The analysis of the fluorescence data was conducted using two different strategies: a conventional JIP test and a novel machine learning-assisted anomaly-detection approach. Our results suggest that exposure to RF-EMFs weakens the plant’s hormetic responses induced by drought treatment, both in terms of the response’s magnitude and its extent. These findings provide further evidence supporting the hypothesis that RF-EMFs interfere with plant stress responses.

## 1. Introduction

When Heinrich Hertz (1857–1894) achieved the first radio signal transmission in 1886, he considered it merely a successful experiment that would prove Maxwell’s theoretical hypothesis of an “electromagnetic wave”. The practical potential of his invention, according to Hertz, was non-existent. Fast forward 150 years, and it is difficult to imagine our world today without the billions of wirelessly connected devices participating in all of our daily activities, large and small. Every wireless device, every cell phone, and every wireless router blankets its surroundings with an invisible electromagnetic field. These electromagnetic fields, known as radiofrequency electromagnetic fields (RF-EMFs), typically have frequencies between 100 kHz and 300 GHz [1]. While there are natural sources of electromagnetic fields in these frequency ranges, such as cosmic rays or lightning in the Earth’s ionosphere, they differ significantly from anthropogenic RF-EMFs in intensity, frequency, and polarization [2]. It could be said that the extraordinary level of RF-EMFs that we are experiencing today is a phenomenon unprecedented in world history and one for which all living organisms are evolutionarily equally ill-prepared. The physiological effects of RF-EMFs on the biosphere are therefore difficult to predict, and their deleterious effects cannot be ruled out [1].

Plants are exposed to RF-EMFs in their environment, much like other organisms. A number of studies have demonstrated various adverse effects of RF-EMFs on plants. The exposure of duckweed (*Spirodela polyrhiza*) to RF-EMFs results in growth retardation and developmental abnormalities [3]. Similar RF-EMF-induced growth inhibition has also been observed in mung bean (*Vigna radiata*), bush rose (*Rosa hybrida*), and radish (*Raphanus sativus*) [4,5,6]. In tomato (*Lycopersicon esculentum*), the rapid transcript accumulation of at least five genes belonging to the calcium-dependent stress response pathway was detected after exposure to RF-EMFs [7]. The induction of reactive oxygen species (ROS), another typical plant defense response to stress, was also detected in RF-EMF-exposed mung bean (*Vigna radiata*) [4]. In addition, numerous other impacts of RF-EMFs on photosynthesis, gene expression, protein production, and secondary metabolism in plants have also been reported [8,9,10,11]. The causes of the adverse effects of RF-EMFs on plants are still poorly understood. The number of studies is still too small, and only a few plant species have been investigated. The RF-EMF frequencies, durations, and intensities investigated in these studies vary widely, making their results difficult to compare [12]. It is also not clear by which mechanisms RF-EMFs affect plants. Many mechanisms of action have been proposed, but none h been clearly demonstrated [1].

A major knowledge gap arises from the fact that most RF-EMF plant studies are conducted under strictly controlled conditions with relatively short exposure times (only hours to a couple of days) [12]. This is a significant deviation from real field conditions where plants are constantly exposed to RF-EMFs and, at the same time, to other environmental factors (sun, wind, temperature fluctuations, insects, etc.). Previous studies have often neglected the role of external stress factors on RF-EMF–plant interactions. In our previous research [13], we have made a surprising discovery of a previously unknown influence of RF-EMFs on the plant stress response. In essence, our experiments showed that RF-EMFs per se cause little stress to plants under stress-free greenhouse conditions but disrupt the normal stress responses of plants to light stress, resulting in plants being more sensitive to light stress in the field.

In our toolbox for analyzing and quantifying stress in plants, one of the most widely used methods is the analysis of the fast light-induced fluorescence transient rise kinetics. When a dark-adapted leaf is exposed to light, chlorophyll fluorescence intensity rapidly increases, following a characteristic polyphasic pattern known as the OJIP curve [14]. This fluorescence rise reflects key aspects and subprocesses of Photosystem II’s photochemical activity and electron transport [15]. The OJIP analysis has gained widespread recognition as a powerful tool for studying plant photosynthesis and stress due to its ease of measurement and its high sensitivity to physiological conditions and environmental factors [16,17,18,19,20,21]. Recent technological advancements, such as the integration of imaging techniques [22,23,24] and the application of machine learning algorithms [25,26,27,28], have significantly enhanced the capabilities of OJIP analysis, further strengthening its role in plant research.

In this present study, we aim to perform an in-depth analysis of lettuce’s (*Lactuca sativa*) fast chlorophyll fluorescence rise kinetics under prolonged RF-EMF exposure in combination with drought stress. The results of the present analysis will provide valuable insights into the question of whether RF-EMFs interfere with plant responses to drought stress and, if so, the nature of such interference.

## 2. Results

### 2.1. Overview of Experiments and Data-Analysis Strategies

Three independent experiments were conducted in July and August 2024. In each experiment, lettuce plants were examined, which were divided into four groups of 9 to 10 plants each:

- Group ED: Plants that were exposed to RF-EMFs and then subjected to drought stress
- Group E: Plants that were exposed to RF-EMFs and watered regularly.
- Group D: Plants that were not exposed to RF-EMFs, then subjected to drought stress
- Group Control: Plants that were not exposed to RF-EMFs and were watered regularly.

The fast chlorophyll fluorescence rise kinetics of all plants were measured using the Open FluorCam FC 800-O/1010 imaging system. Measurement points were extracted from the fluorescence image as described in Section 4. In general, 50 to 100 measurement points were recorded from each plant, depending on its size. In total, 7057 points were collected, as shown in Table 1. Each measurement point includes a fast chlorophyll fluorescence rise (OJIP) curve and a set of 31 different parameters derived from the conventional JIP tests. Detailed descriptions of these parameters are provided in the Supplementary Materials (see Table S1).

Two data-analysis strategies were implemented: a conventional approach and a novel Machine Learning (ML)-based anomaly detection approach. Data from each experiment were analyzed independently.

Strategy 1: Conventional approach

- Step 1: Perform the JIP test on all measurements
- Step 2: Compare the OJIP parameters of the control group and group D to determine the response pattern of lettuce to drought stress (i.e., which OJIP parameters are indicative of a drought treatment). The criteria for the determination of drought indicators are the (1) statistical significance, (2) effect size, and (3) direction of the effect.
- Step 3: Perform a two-way ANOVA on the data of the relevant parameters from all groups to determine if there is a statistically significant interaction between RF-EMF exposure and drought treatment.
- Step 4: Similarly, compare the OJIP parameters of the Control group with group E to determine the effect of RF-EMF exposure on plant OJIP behavior.

Strategy 2: Recently, we have developed a novel ML-based anomaly-detection approach [28]. The underlying principle of this methodology is predicated on the assumption that the response of a plant to stress is not spatially uniform; rather, certain parts of the plant may appear to maintain a healthy OJIP pattern. When the data is averaged across the entire organism, such ostensibly “normal” results render it more challenging to identify the small differences between stressed and control plants. To discern and eventually filter out these data points, an anomaly-detection approach is necessary. The workflow is as follows:

- Step 1: Assume that all of the data points in the control group represent a “normal” OJIP pattern and that the drought treatment would have resulted in the emergence of several “anomalous” OJIP points in group D.
- Step 2: Build an ML-based anomaly detection model via training with OJIP kinetic data from the control group and cross-validate with data from both the control group and group D. Detailed descriptions of model training and cross-validation are provided in Section 4 and in the Supplementary Materials.
- Step 3: Apply this established model to detect all “anomalies” from the data of all four groups. Analyze the distribution of “anomalies”.
- Step 4: Perform the conventional JIP test on all “anomalies” and “normal” measurements.
- Step 5: Compare the OJIP parameters of the control group to the “anomalies” of group D to determine the response pattern of lettuce to drought stress.
- Step 6: Compare the OJIP parameters of the control group to the “anomalies” of group E to determine the response pattern of lettuce to RF-EMF exposure.
- Step 7: Compare the OJIP parameters of the control group to the “anomalies” of group ED to determine the response pattern of lettuce to the combined treatment of drought and RF-EMF exposure.
- Step 8: Compare the “anomalies” of group D and group ED with respect to the previously identified drought indicators to assess the effect of RF-EMF exposure on the drought response.

### 2.2. Conventional Approach Using a Two-Way ANOVA Revealed Significant, Albeit Small, Interaction Effects Between the Drought Treatment and RF-EMF Exposure

Lettuce plants were grown in an RF-EMF-free environment until they were three weeks old. The OJIP curves were measured at the start of treatment (null measurement) and twice within a period of ten days. Null measurements show no statistically significant difference between groups. To investigate the plants’ response to drought stress, groups D and ED were exposed to four hours of no irrigation on warm, sunny days (weather data are available in the Supplementary Material Figure S2).

**Figure 1:**
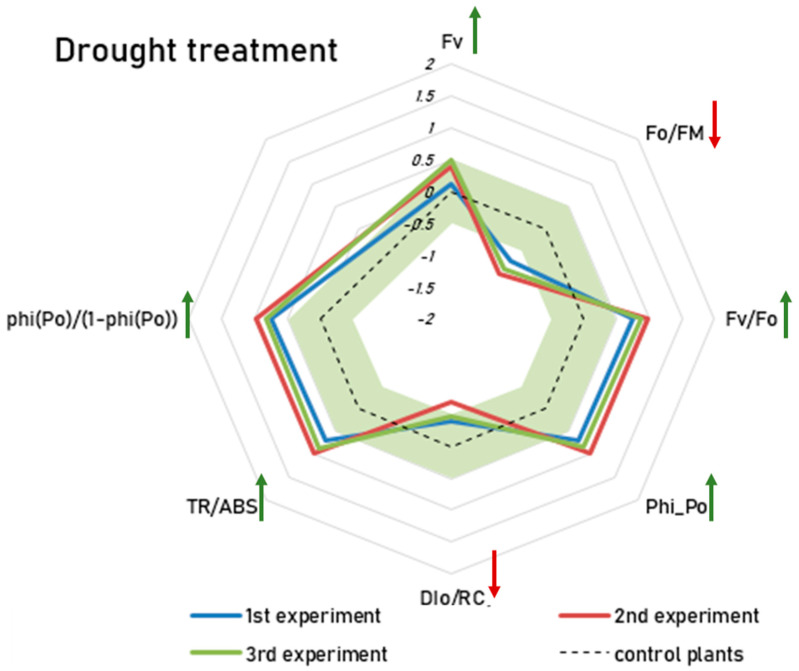
JIP test on the drought treatment of group D (drought treatment): blue line—experiment 1, red line—experiment 2, green line—experiment 3. The radar plot shows the effect sizes (Cohen’s d), which indicate the standardized difference between the group D and control plants. The dotted line represents the control plants where all values are set to zero. The green area represents negligible effects with Cohen’s d values between −0.5 and 0.5. Only differences outside this range are considered meaningful. Group D consistently has higher F_v_, F_V_/F_O_, F_V_/F_M_ (ϕP_O_), TR_O_/ABS, and ϕ(Po)/(1 − ϕP_O_) and lower DI_O_/RC and F_O_/F_M_ (all *p* < 0.05). The arrows indicate the direction of the effect (green = increase, red = decrease).

We closely examined all OJIP parameters of group D to identify which biochemical processes of photosynthesis were affected by drought. The criteria that we used to select the parameters affected by drought were their significant difference from the pattern of the control plants and the consistency of the effect’s direction. Seven parameters were identified that consistently showed statistically significant differences between the control group and group D, with the direction of the difference remaining stable across all three experiments. These parameters are used as indicators of the drought response. Figure 1 shows the effect sizes (Cohen’s d) for the drought response parameters from all three experiments. Cohen’s d represents the standardized difference between two groups, with values of 0.2, 0.5, and 0.8 indicating small, medium, and large effects, respectively.

- FV = variable Chlorophyll a fluorescence—weak effect size (Cohen’s d = 0.12–0.49)
- Phi_Po (Fv/Fm) = maximum quantum yield for primary photochemistry—medium/strong effect size (Cohen’s d = 0.7–0.97)
- DIo/RC = dissipation flux per reaction center—weak/medium effect size (Cohen’s d = −0.38–−0.69)
- Fo/FM = quantum yield of energy dissipation—medium/strong effect size (Cohen’s d = −0.72–−0.99)
- Fv/Fo = potential photochemical efficiency and is also an indicator of the size and number of active photosynthetic reaction centers—medium/strong effect size (Cohen’s d = 0.75–0.99)
- TR/ABS = energy flux ratio between trapping and absorption—medium/strong effect size (Cohen’s d = 0.71–0.98)
- phi(Po)/(1 − phi(Po)) = the probability that the absorbed photon will be trapped by RC—medium/strong effect size (Cohen’s d = 0.74–0.97) [29,30].

Changes in these seven parameters generally indicate an improved photosynthetic efficiency following short-term drought treatment, with the exception of DI_0_/RC and F_0_/F_m_, which are related to dissipation.

In the next step, after identifying drought-relevant parameters, a two-way ANOVA was performed across all groups for these seven parameters to test for an interaction between drought and RF-EMF exposure and to assess the drought stress response of RF-EMF-exposed plants. The results of the two-way ANOVA are presented in Table 2.

The results show that there was a significant interaction between RF-EMF exposure and drought treatment for the previously identified parameters. A closer look at the direction of the effect showed that except DIo/RC in the 1st experiment, RF-EMFs consistently reduced the response of lettuce plants to drought stress. The ED group always showed a weaker response to RF-EMF exposure compared to the D group. In our study, however, the partial eta-squared effect size (η^2^p) for the drought–EMF interaction indicated very small effects (η^2^p between 0.003 and 0.016). For reference, a η^2^p value of 0.01 indicates a small effect, 0.06 a medium effect, and 0.14 a large effect.

**Figure 2:**
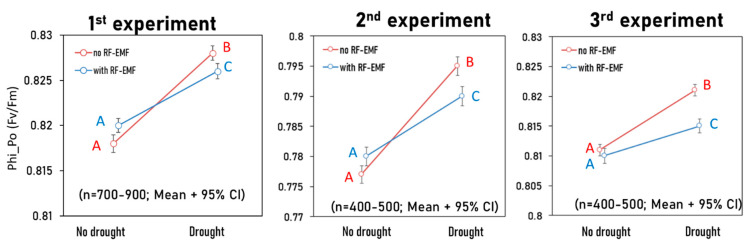
Interaction plot of the parameter PhiPo_Po (Fv/Fm): blue line—control group and group D; red line—group E and group ED. Differences in the letters indicate significant differences, with distinct letters representing statistically significant differences (*p* < 0.05). The interaction between drought and drought + EMF shows a significant effect across all three experiments (*p* < 0.05).

As an example, we show the effects of RF-EMF exposure and drought treatment on the Fv/Fm parameter (Figure 2). The red line represents the No-RF-EMF variant, including both control and drought conditions. The increase reflects the natural hormetic response of lettuce to short drought stress (plant hormetic responses to drought are further discussed in Section 3.1). RF-EMF exposure significantly reduced this natural stress response (blue line). For other parameters, the graphs follow the same pattern.

Another point of interest is the effect of RF-EMF exposure alone on photosynthetic efficiency.

**Figure 3:**
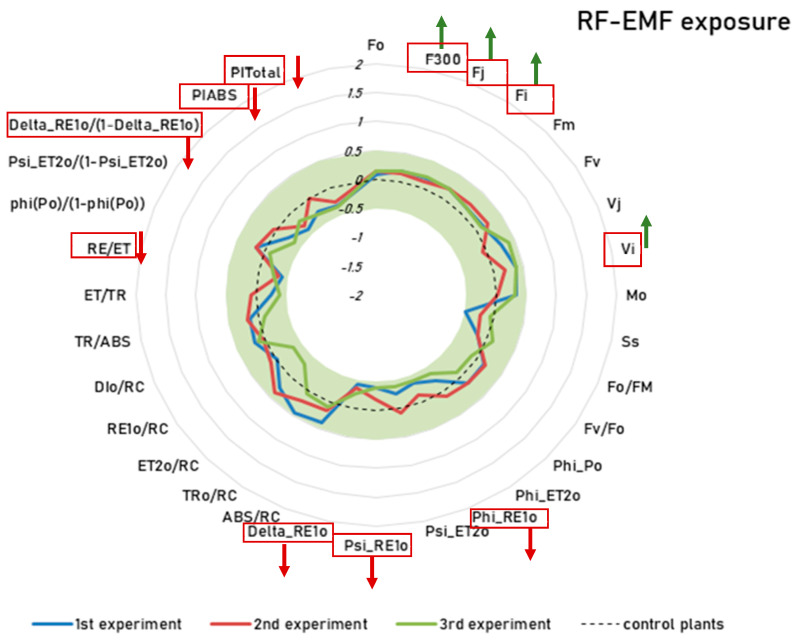
JIP test on the group E (RF-EMF treatment) and the control group: blue line—experiment 1, red line—experiment 2, green line—experiment 3. The radar plot shows the effect sizes (Cohen’s d), which indicate the standardized difference between the group E and the control plants. The dotted line represents the control plants where all values are set to zero. The green area represents negligible effects with Cohen’s d values between −0.5 and 0.5. Only differences outside this range are considered meaningful. Group E consistently had higher F_300_, F_j_, F_i_, and V_i_ (P_O_) and lower Phi_RE1_0_, Psi_RE10, Delta_RE1_0_, RE/ET, Delta_RE1_0_/(1 − Delta_RE1_0_), PI_abs_, and PI_total_ (all *p* < 0.05). These parameters are outlined in red with arrows indicating the direction of the effect (green = increase, red = decrease).

The JIP test analysis, in the comparison between group E (EMF) and the control group, shows that the plants exposed to RF-EMFs consistently had significantly higher values for F_300_, F_j_, F_i_, and V_i_, while showing significant lower values for PI_ABS_, PI_Total_, delta_Re1o, RE/ET, Delta_RE1o, Psi_RE1o, and Phi_RE10 across all three experiments (Figure 3). Detailed descriptions of these parameters are provided in the Supplementary Materials. However, as shown in Figure 3, the Cohen’s d value never exceeded the threshold for a small effect (±0.5). In short, a comparison between the control group and group E (RF-EMF) shows that RF-EMF exposure alone leads to a significant reduction in photosynthetic efficiency, albeit with a small effect size. This finding is consistent with the results reported in reference [13], which found that prolonged exposure to RF-EMFs caused a significant and systemic reduction in photosynthetic efficiency (see Section 3.2 for further discussion).

### 2.3. Novel Machine Learning (ML)-Based Anomaly-Detection Approach Also Confirms the Aforementioned Interaction Effects Between Drought Treatment and RF-EMF Exposure, but with a Larger Effect Size

**Figure 4:**
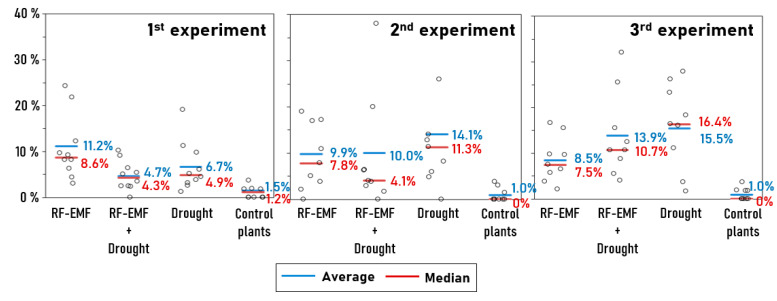
Distribution of “anomalies” in all four groups over three experiments. Each dot represents the percentage of “anomalies” of an individual plant.

By implementing the proposed strategy of ML-based anomaly detection, we were able to identify many “anomalies”, i.e., measuring points for which the OJIP curves differed significantly from the pattern of the control plants. The results are summarized in Table 3 and Figure 4. It is notable that the average percentage of “anomalies” per plants was always higher with group D (drought treatment only) than with group ED (drought treatment and RF-EMF exposure) across all three experiments, regardless of whether the mean or median values were used for comparisons.

**Figure 5:**
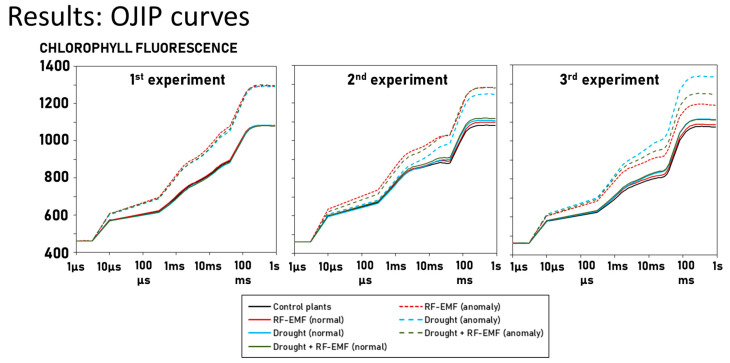
Averaged OJIP curve of “anomalies” (dotted lines) and “normal” (solid lines) measurements. “Anomalies” from the control plants are not shown because they represent only about 1% of this group.

Figure 5 shows the averaged OJIP curve of “anomalies” and “normal” measurements. In all cases, “anomalies” tend to give higher chlorophyll fluorescence in all phases of the OJIP curve. As expected, the “normal” curves from all groups are very similar to each other.

In the next step, to determine the responses of the plants to drought treatment, RF-EMF exposure, and their combination, we compared the “anomalies” of groups D and E and ED with the “normal” measurements of the control plants using the conventional JIP test. These “anomalies” are found to be significantly different in nature.

**Figure 6:**
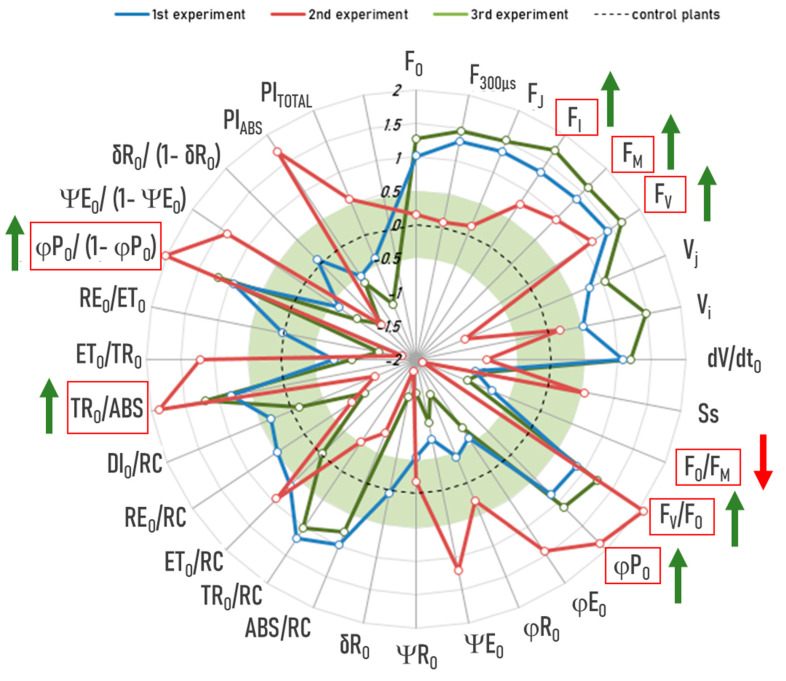
JIP test on the “anomalies” of group D (drought treatment): blue line—experiment 1, red line—experiment 2, green line—experiment 3. The radar plot shows the effect sizes (Cohen’s d), which indicate the standardized difference between the “anomalies” and the control plants. The dotted line represents the control plants where all values are set to zero. The green area represents negligible effects with Cohen’s d values between −0.5 and 0.5. Only differences outside this range are considered meaningful. The “anomalies” of group D consistently had higher F_I_, F_M_, and F_V_, lower F_O_/F_M_, and higher F_V_/F_O_, F_V_/F_M_ (ϕP_O_), TR_O_/ABS, and ΨP_O_ Po/(1 − ϕP_O_) (all *p* < 0.05). These parameters are outlined in red with arrows indicating the direction of the effect (green = increase, red = decrease).

Group D’s “anomalies”: We performed a JIP test on the “anomalies” of Group D (drought treatment) and compared them with the control plants in the same experiment. The substantial quantity of measurements and parameters that are subject to analysis can result in a considerable number of statistically significant yet negligible differences. Thus, we also calculated the effect size (Cohen’s d) to evaluate the magnitude of the difference [31]. Cohen’s d indicates the standardized difference between two compared groups. The conventional interpretation of Cohen’s d is as follows: d = 0.2—small effect (the difference is small and may be difficult to detect); d = 0.5—medium effect (the difference is noticeable); d = 0.8—large effect (the difference is substantial) [31]. In our study, differences were considered meaningful only if (1) they were statistically significant (*p* < 0.05) and (2) Cohen’s d was either greater than 0.5 or less than −0.5. Using these criteria, we could show that the “anomalies” of group D consistently have higher F_I_, F_M_, and F_V_, lower F_O_/F_M_, and higher F_V_/F_O_, F_V_/F_M_ (ϕP_O_), TR_O_/ABS, and ϕPo/(1 − ϕP_O_) across all three experiments (Figure 6). Together, these differences indicate a more efficient primary photochemistry (so-called trapping, i.e., the capture of excited energy absorbed by the PSII antennae for the reduction of Pheo and Q_A_) in Group D’s “anomalies” compared to the control plants.

**Figure 7:**
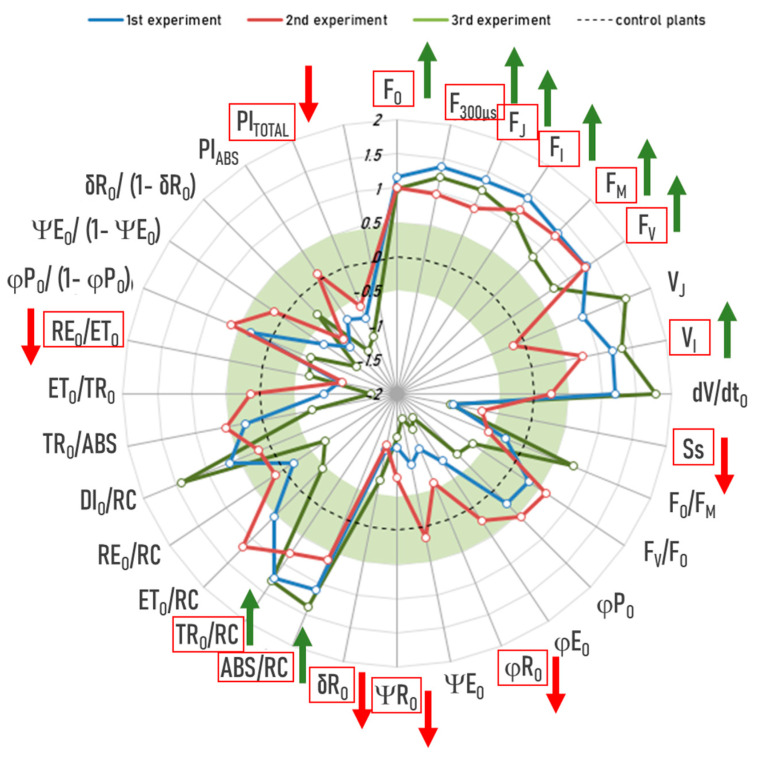
JIP test on the “anomalies” of group E (exposed to RF-EMFs): blue line—experiment 1, red line—experiment 2, green line—experiment 3. The radar plot shows the effect sizes (Cohen’s d), which indicate the standardized difference between the “anomalies” and the control plants. The dotted line represents the control plants where all values are set to zero. The green area represents negligible effects with Cohen’s d values between −0.5 and 0.5. Only differences outside this range are considered meaningful. The “anomalies” of group E consistently had higher F_O_, F_300µs_, F_J_, F_I_, F_M_, and F_V_; higher V_I_, lower Ss, and lower ϕRo, ΨRo, and δRo; higher ABS/RC and TR_O_/RC; and lower RE_O_/ET_O_ and lower PI_TOTAL_ (all *p* < 0.05). These parameters are outlined in red with arrows indicating the direction of the effect (green = increase, red = decrease).

Group E’s “anomalies”: Similarly, we performed a JIP test on the “anomalies” of Group E (exposed to RF-EMFs) and compared them with the control plants. Our results indicate that the “anomalies” of group E consistently had higher F_O_, F_300µs_, F_J_, F_I_, F_M_, and F_V_; higher V_I_, lower Ss, and lower ϕRo, ΨRo, and δRo; higher ABS/RC and TR_O_/RC; and lower RE_O_/ET_O_ and lower PI_TOTAL_ across all three experiments (Figure 7). Taken together, these differences indicate a lower density of active reaction centers, lower energy flux, and lower quantum efficiency of end-acceptor reduction on the Photosystem I side, in the Group E’s “anomalies” compared to the control plants.

**Figure 8:**
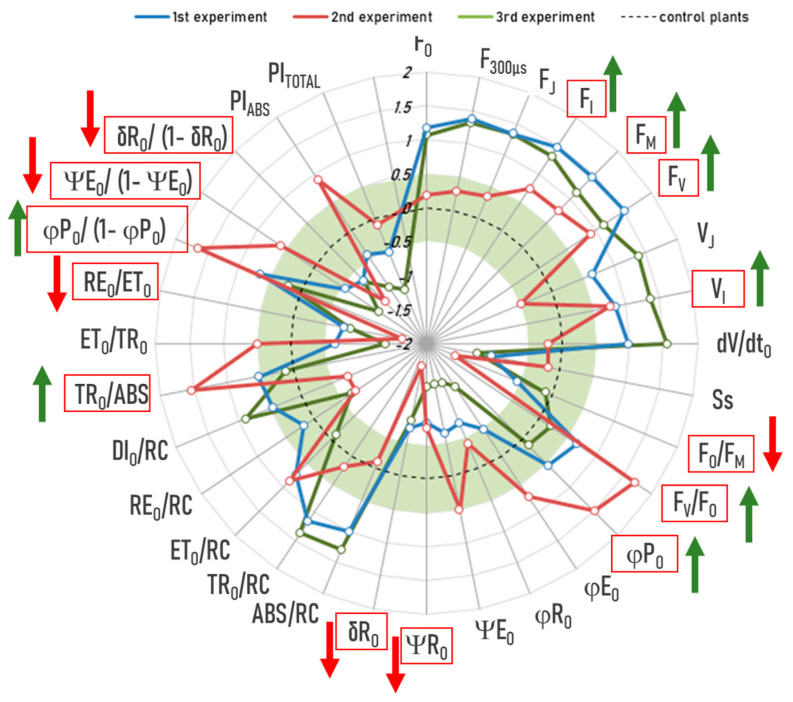
JIP test on the “anomalies” of group ED (exposed to RF-EMFs and subjected to drought treatment): blue line—experiment 1, red line—experiment 2, green line—experiment 3. The radar plot shows the effect sizes (Cohen’s d), which indicate the standardized difference between the “anomalies” and the control plants. The dotted line represents the control plants where all values are set to zero. The green area represents negligible effects with Cohen’s d values between −0.5 and 0.5. Only differences outside this range are considered meaningful. The “anomalies” of group ED consistently had higher F_I_, F_M_, and F_V_; higher V_I_; lower ΨRo and δRo, and lower RE_O_/ET_O_, ΨEo/(1 − ΨE_O_), and δRo/(1 − δRo) and, in 2/3 experiments, lower F_O_/F_M_, higher F_V_/F_O_ and F_V_/F_M_ (ϕP_O_), and higher TR_O_/ABS and ϕPo/(1 − ϕP_O_) (all *p* < 0.05). These parameters are outlined in red with arrows indicating the direction of the effect (green = increase, red = decrease).

Group ED’s “anomalies”: An interesting picture emerged when we analyzed the OJIP parameters of the “anomalies” of group ED (exposed to RF-EMFs and subjected to drought treatment) and compared them with those of the control plants (Figure 8). Our results indicate that the “anomalies” of group E consistently also had higher F_I_, F_M_, and F_V_. On one side, they had higher V_I_, lower ΨRo and δRo, lower RE_O_/ET_O_, and lower ΨEo/(1 − ΨE_O_) and δRo/(1 − δRo) across all three experiments. Similar to the “anomalies” of Group E, these differences indicate lower energy flux and lower quantum efficiency of end-acceptor reduction on the Photosystem I side. On the other hand, in Experiment 1 and 2, Group ED’s “anomalies” had lower F_O_/F_M_, higher F_V_/F_O_ and F_V_/F_M_ (ϕP_O_), and higher TR_O_/ABS and ϕPo/(1 − ϕP_O_) similar to Group D’s “anomalies”. A similar tendency was also observed in Experiment 3, albeit with modest effect sizes. Together, they point to a more efficient primary photochemistry. In other words, the “anomalies” of Group ED exhibit both the characteristics of the Group E’s “anomalies” and those of Group D.

Comparison of the Groups D and ED’s “anomalies” with respect to the previously identified drought indicators: The next step was to compare the early plant response to drought treatments in group D (drought treatment) and group ED (exposed to RF-EMFs and subjected to drought treatment). We compared the eight OJIP parameters that characterized group D’s responses to drought: F_I_, F_M_, F_V_, F_O_/F_M_, F_V_/F_O_ F_V_/F_M_ (ϕP_O_), TR_O_/ABS, and ϕPo/(1 − ϕP_O_). The results are summarized in Table 4. Our results overwhelmingly indicate that group D responded much more strongly to the drought treatment than group ED, especially in experiments 2 and 3.

In summary, our results demonstrate that “anomalies” in group D are more prevalent than in group ED, and the former also exhibits more substantial changes in regard to typical drought indicators than the latter. Taken together, they indicate that group D responded much more strongly to drought treatment than group ED.

### 2.4. Measurements of Fresh and Dry Matter Reveal the Negative Effects of Drought and RF-EMF Exposure on Plant Growth

**Figure 9:**
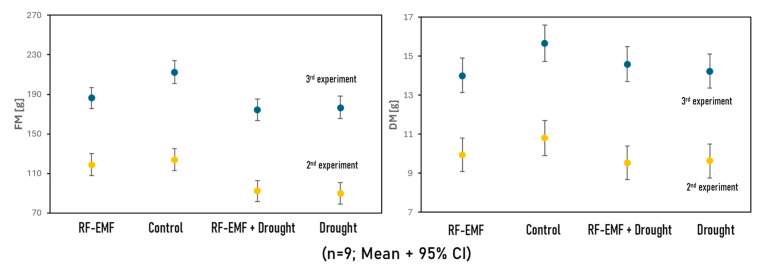

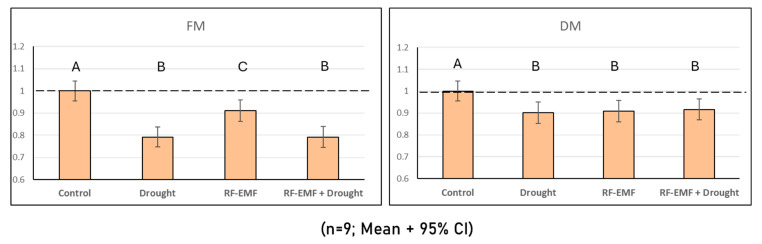
(Above) Fresh matter (FM, left) and dry matter (DM, right) in grams [g] for lettuce plants from experiments 2 and 3. (Below) All FM and DM values were normalized according to the values of the corresponding control groups. The normalized values from the two experiments were then pooled and subjected to a one-way ANOVA. Differences in the letters indicate significant differences, with distinct letters representing statistically significant differences (*p* < 0.05).

In this study, the fresh matter (FM) and dry matter (DM) of lettuce plants from experiments 2 and 3 were assessed. Fresh plants were initially weighed to determine their fresh weight, then placed in pre-weighed paper bags, and dried in an oven at 105 °C for 24 h. After drying, the samples were reweighed to calculate the dry matter (DM). Figure 9 displays the results of the FM and DM determinations from Experiments 2 and 3. In both experiments, the FM and DM of the control group were significantly higher than those of all the other groups (D, E, and ED), suggesting that RF-EMF exposure and drought treatment both negatively impacted plant growth. Interestingly, although the fresh matter of group E was still significantly greater than that of group ED, there was no difference in the dry matter (DM) of the two groups.

## 3. Discussion

### 3.1. Plant’s Hormetic Responses to Drought Stress

Drought stress is a critical environmental factor that significantly affects plant growth, development, and productivity. It occurs when water availability falls below the optimal level required for normal physiological functions, triggering a cascade of morphological, physiological, and biochemical changes [32]. Prolonged drought conditions can severely disrupt plant metabolism, reduce biomass accumulation, and, in extreme cases, lead to plant mortality [33].

Interestingly, in our experiments, group D’s drought-treated plants exhibited *higher* photosynthetic performance than control plants. This counterintuitive finding can be explained by the fact that chlorophyll fluorescence measurements were taken less than 24 h after the initiation of the drought treatment. Thus, the observed effects represent the early responses of plants to a relatively low level of drought. Our case can be very well explained by a well-documented phenomenon in plant biology known as hormesis—the early beneficial effect of low-dose stress followed later by a negative impact at higher stress levels [34]. Such a response is often viewed as an overcompensation mechanism by plants to a disruption in homeostasis [35]. Hormetic effects have been observed across a wide range of species, environmental stressors, and physiological processes, including photosynthesis [36]. For example, studies have demonstrated that low concentrations of metal ions, salt, and herbicides can enhance the photochemistry of Photosystem II. The roles of reactive oxygen species (ROS) and non-photochemical quenching (NPQ) are thought to be central to these adaptive responses [37].

**Figure 10:**
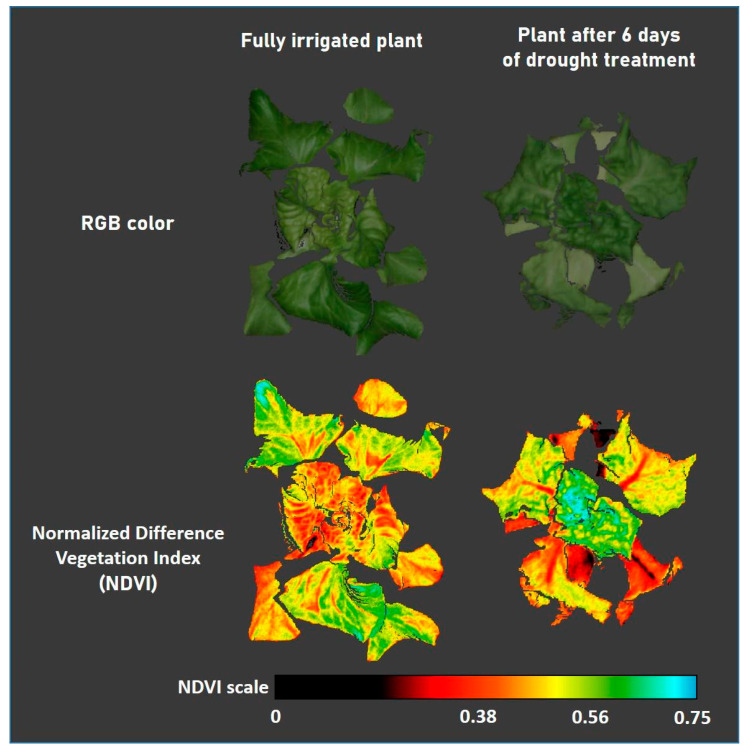
Spatially heterologous plant responses to drought treatment. The image shows the top view of plant 3D-scans with the PlantEye system (Phenospex), depicting the plant’s RGB color and its Normalized Difference Vegetation Index (NDVI). On the left is a 4-week-old lettuce plant that has been watered daily; on the right is another plant of similar age that has not been watered for 6 days. The hormetic effect in the drought-treated plant is evident from the very high NDVI values in the leaves in the middle (author’s own unpublished data).

In our experience, hormetic reactions are very common in lettuce. Interestingly, they can persist in certain plant parts even after prolonged drought stress. We often observe that when lettuce plants are subjected to drought, the old leaves at the periphery wilt quickly and show clear symptoms of stress, such as much lower photosynthetic performance, discoloration, and even desiccation. The young leaves in the center, on the other hand, remain relatively fresh for much longer and actually maintain a higher level of vitality (Figure 10). We postulate that this is a natural strategy of lettuce to cope with drought stress.

### 3.2. Effects of RF-EMF Exposure on Plant Health and Plant Responses to Stress

In this study, we chose drought stress as an additional stress factor because, in the near future, we can expect both a substantial increase in the use of wireless technologies and a significant rise in extreme weather events, such as drought, as a result of climate change. These two factors, along with their potential interactions, could have considerable impacts on plant health and development. Our findings suggest that prolonged exposure to RF-EMFs has a discernible, albeit small, negative impact on plant health. Using our novel ML-based anomaly-detection approach, we identified “anomalies” in approximately 10% of measurement points in Group E (exposed to RF-EMFs). A further analysis of these “anomalies” consistently revealed lower densities of active reaction centers, reduced energy flux, and decreased quantum efficiency in end-acceptor reduction on the Photosystem I side compared to control plants. These results closely align with our prior field experiments conducted between 2022 and 2023, where we also observed the adverse effects of RF-EMF exposure under outdoor conditions [13]. The observed patterns of RF-EMF effects appear to be in good agreement between the two studies. Most notably, a decline in δR_O_, the quantum yield of end-acceptor reduction at the PSI side, and an increase in RC/ABS, the effective antenna size, have been consistently observed in RF-EMF-exposed plants in all involved experiments. This suggests that these two subprocesses may be pivotal in comprehending the RF-EMF effects on plants. In the future, exploring the potential impacts of RF-EMFs on these parts of the photosynthetic reactions warrants further investigation.

Our findings strongly indicate that prolonged exposure to RF-EMFs weakens plant hormetic responses to drought stress in both the extent and magnitude. A two-way ANOVA revealed consistent interaction effects between RF-EMF exposure and drought treatment, showing that plants exhibited significantly weaker responses under RF-EMF exposure. The utilization of an ML-based anomaly-detection approach yielded a similar conclusion, albeit with additional nuances. We observed a consistently lower percentage of “anomalies” in group ED (exposed to RF-EMFs and subjected to drought treatment) compared to group D (drought treatment). Moreover, the drought responses of group ED’s “anomalies” were significantly weaker than those of Group E in all metrics. In our previous publication, we reported that RF-EMF exposure disrupts plant responses to light stress by inhibiting the expression of stress-related genes and weakening NPQ, making plants more susceptible to light stress. Our current findings further reinforce our hypothesis that RF-EMF exposure interferes with general plant stress responses.

The prospect of potential interference of RF-EMFs with plant stress responses naturally raises the question of whether RF-EMF exposure could affect plant growth. Although this question was not directly addressed in these studies, the results of dry weight quantification provided intriguing insights. We observed that plants in group E had significantly lower dry weights than those in the control group, indicating that RF-EMF exposure does affect plant growth under the conditions of the study. Conversely, no discernible distinction was observed between group D and group ED; both exhibited significantly diminished dry weights in comparison to the control plants. This finding indicates that RF-EMF exposure did not exacerbate the severity of drought stress, despite its interference with drought responses.

### 3.3. Advantages and Limitations of Our Machine-Learning-Assisted Data-Analysis Approach

In this study, we utilized a novel ML-based anomaly-detection methodology for the analysis of fluorescence data. This approach is based on the assumption that stress responses are highly heterogeneous within a plant and that the conventional averaging approach is insufficient for small changes or when the majority of the plant is still unaffected [28]. The merit of our novel approach is demonstrated in this study in the case of group E (plants exposed to RF-EMFs). Our previous research has shown that the discernible effects of RF-EMFs are typically only observable after a period of continuous exposure lasting three to four weeks [13]. In the present study, the OJIP parameters of group E exhibited, on average, negligible variation in comparison to the control plants following one week of RF-EMF exposure. A conventional approach would have concluded that such a short period of exposure has no significant effect on plant health and plant photosynthesis. However, using our novel ML-assisted approach, we could however clearly show that RF-EMF exposure leads to the appearance of a small but consistent number of “anomalies” for which photosynthetic performance is lower than the control group, indicating a debilitating RF-EMF effect.

On the other hand, due to the “black box” nature of ML models, for which conclusions are derived solely from data rather than from a verifiable underlying theory, it is imperative to exercise greater caution when assessing the appropriateness of such an approach and the validity of its interpretation. These considerations must be determined prior to data analysis and made transparent. The selection of training and validation data is of paramount importance. In this study, data from the control group was selected to establish the “normal” OJIP pattern, and data from both the control group and group D (drought treatment) was selected for model validation. These choices are substantiated by the following rationale:

- The training, validation, and test datasets were all derived from plants of the same age, cultivar, and cultivation history, all of which were cultivated prior to the experiment under identical conditions. Consequently, the “anomalies” identified by the model are unlikely to be attributable to these factors and only to the treatment of plants. Otherwise, it would be difficult to correctly interpret the results.
- In order to train the model to recognize the “normal” OJIP pattern, data from the control group was used. This approach was based on the underlying assumption that OJIP patterns measured from the control group plants were relatively homogeneous. This assumption is reasonable given that the control group plants were relatively young and well-tended [28].
- Nonetheless, subtle variations in plant individuals are invariably present. It is imperative that the model is adequately generalized so as to avoid identifying such minute differences as “anomalies” (i.e., the model identifies a measurement as “anomalous” simply because it was taken with a different plant than from the training data, a phenomenon often referred to as model overfitting). To guarantee this, a number of plants from the control group were incorporated into the validation data.
- Among group E, group D, and group ED, drought treatment is the only one that has been definitively linked to alterations in the OJIP pattern, thereby giving rise to what are referred to as “anomalies.” Consequently, it is logical to utilize, exclusively, the data from group D in the validation dataset.

Conversely, these rationale also define the limitations of the model. It is, for example, impossible to apply a model from one experiment to the others, as they took place at different times of the year and under different conditions. Therefore, anomaly-detection models have to be established for each experiment individually. Our approach would also be ill-suited for the analysis of old plants or plants that have previously experienced stress because the underlying assumption that OJIP patterns measured from control group plants were relatively homogeneous could no longer be guaranteed.

In the present study, only the OJIP characteristics of the “anomalies” were examined. However, their spatial distribution pattern itself constitutes a valuable piece of information that could add another layer of depth to the already substantial amount of information that can be extracted from OJIP measurements. It would be of great interest to explore this approach in future studies.

### 3.4. Outlook

The results of this study lend further credence to the hypothesis that RF-EMFs potentially interfere with normal plant stress responses. Admittedly, our studies to date have focused exclusively on one plant species and one identical RF-EMF condition; therefore, any generalized conclusion is unwarranted at this stage. Nevertheless, our world is currently experiencing rapid transformations. In the coming years, it is not inconceivable that the entire planet could be covered by mobile signals, with increasingly greater RF-EMF intensities and ever-expanding frequency spectra. Concurrently, climate change is manifesting at an alarming rate, resulting in the rapid alteration of numerous plant species’ habitats and leading to the ever-increasing frequency of extreme weather events. A potential weakening of plant stress responses at a time when they are facing unprecedented challenges from climate change would spell disaster for our ecosystem and our food security. Therefore, we see an urgent need for more studies on the effects of RF-EMF exposure on plants, in particular on plant stress responses, to fill the still very large gaps in our knowledge on this topic.

## 4. Material and Methods

### 4.1. Plant Cultivation

*Lactuca sativa* (cultivars Briweri and Lucinde) was grown in soil pots under greenhouse conditions. The temperature was maintained between 19 °C and 23 °C with a relative humidity of 50–60%. The RF-EMF intensity in the greenhouse was negligible. At the beginning of the experiment, the *Lactuca sativa* plants were divided into four groups of 9–10 plants each. The three-week-old plants were then placed in the Forschungsring e.V. experimental field. The control plants, positioned next to the RF-EMF-exposed area, were shielded from the nearby RF-EMF emitters by a fine-mesh metal fence (mesh size 13 mm, height 120 cm), serving as a reference.

### 4.2. RF-EMF Exposure

We generated artificial electromagnetic fields (frequency ranges: 1880–1900 MHz DECT, 2.4 and 5 GHz WLAN) in a Forschungsring e.V. test field to study potential effects on photosynthetic efficiency.

The RF-EMF was generated by two Wi-Fi routers (Fritzbox 7530) with an integrated DECT base station and two DECT phones (Motorola t412+ in deactivated eco-mode). The DECT phones were in a permanent telephone connection with an additional terminal. The frequency band from 1880 to 1900 MHz is allocated exclusively for DECT (Digital Enhanced Cordless Telecommunications) systems in Europe. This band is primarily used by cordless telephones, typically deployed in residential environments. The 1880–1900 MHz frequency band lies adjacent to bands used for mobile communications (e.g., GSM1800). The RF-EMF radiation was measured using two broadband RF analyzers from Gigahertz Solutions. Radiation in the 1880–1900 MHz and 2.4 GHz frequency ranges, measured with the HF59B high-frequency analyzer (covering 700 MHz to 2.7 GHz), was 8000 μW/m^2^ (peak measurement). The upper Wi-Fi band (5 GHz) measured with the HFW35C high-frequency analyzer (covering 2.4–6 GHz) was 2000 μW/m^2^ (peak measurement).

**Figure 11:**
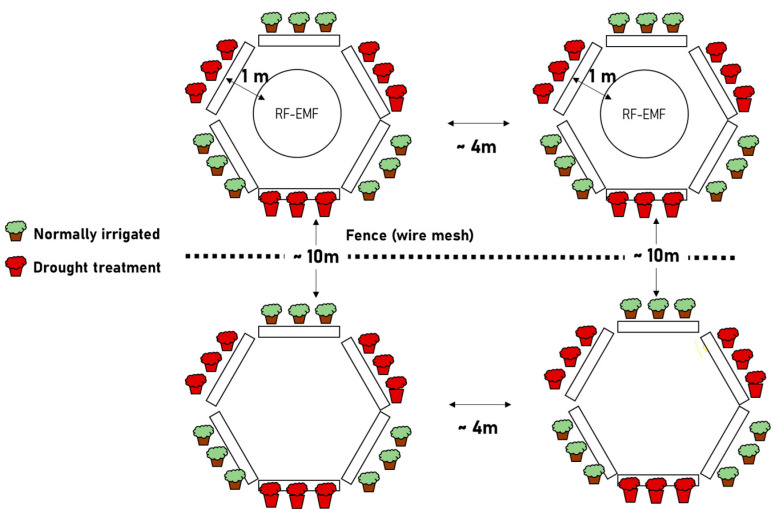
Experimental setup—exposure of lettuce plants to RF-EMF.

The control plants (Figure 11) placed adjacent to the RF-EMF-exposed area were shielded from the adjoining RF-EMF emitters with a fine-mesh metal fence (mesh size 13 mm and height 120 cm) and therefore served as a reference.

The RF-EMF-exposed plants were positioned in a circular arrangement at a distance of one meter from the emitters (see Figure 11). The power flux density was measured and found to be identical for all lettuce specimens. High-frequency analyzers were used to verify the minimal level of RF-EMF exposure experienced by the control groups.

### 4.3. Measurements of Fast Chlorophyll Fluorescence Kinetics

The efficiency of photosynthesis was measured using an Open FluorCam FC800-O/1010 (Photon Systems Instruments, Drásov, Czech Republic) equipped with a fast CCD 735 camera, which allows the measurement of fast fluorescence kinetics (OJIP analysis). Fluorescence transients induced by actinic light or saturating flashes can be imaged with this camera. User-defined protocols determine the timing and amplitude of the actinic irradiance. The system calibration and data processing procedures are described in Supplementary Material, Chapter S1. The data extraction from chlorophyll fluorescence imaging is illustrated in Figure S1, which shows the grid-based segmentation of the plant image. The lettuce plants were measured in a dark room after a 30 min dark adaptation period. From the control group and from the E group, one plant was taken out and measured in alternation. The same procedure was used for the ED and D groups. OJIP parameters were calculated using FlourCam 10 (version 1.0.1.2) software.

### 4.4. Machine-Learning-Assisted Data Analysis

Anomaly detection was performed on the OJIP kinetic data of the measurement points. Each data point generally comprises 51 attributes, which correspond to the 51 time marks in the OJIP curve. We employed DD-SIMCA, a MATLAB-based (version R2024b) tool for data-driven one-class classification, to carry out this task [38]. Anomaly-detection models were established independently for each experiment. The training set comprises data points from six plants in the control group. For all model training, we employed a type I error rate (alpha) of 0.01 and an outlier significance of 0.01. We utilized a “chi-square” type of acceptance area and a “classical” method of estimating the model parameters. The parameters of the model that were subject to optimization were the number of principle components (PCs) and the data-preprocessing method. A total of four data-preprocessing regimes were evaluated in this study: (1) no preprocessing, (2) column-wise centering, (3) scaling by the standard deviation, and (4) column-wise centering and scaling by the standard deviation. The number of PCs was initially set at 2 and subsequently increased incrementally until it became evident that the model was overfitted (see below).

Two datasets were utilized for model validation and assessment. The first validation dataset contained data points from three plants of the control group that differed from those employed during model training. The second validation dataset comprised data points from all plants of group D, which were drought-stressed. As the number of PCs with both validation datasets increased, the percentage of anomalies typically increased concomitantly (refer to the Supplementary Materials for more details Figure S3 and Table S2). Therefore, the optimal model is one that strikes a balance between being sufficiently flexible to not recognize data from the first validation set as anomalies, while still retaining the capability to recognize anomalies in the second validation set.

The following criteria were employed for model assessment and optimization:

- With the training dataset, the percentage of anomalies should be approximately 1%.
- With the validation dataset #1, the percentage of anomalies should be less than 200% of that of the training dataset. The optimization of the model was halted once the percentage of anomalies in the validation dataset #1 surpassed 1000% of that observed in the training dataset.
- With the validation dataset #2, the percentage of anomalies should be as high as possible while still meeting the above criteria.

Optimized models were subsequently employed to detect anomalies in the remaining groups E and ED.

### 4.5. Statistical Analysis

The statistical data analysis (two-way ANOVA and *t*-test) of the JIP-test parameters was conducted using Jamovi (version 2.6.13) and Microsoft Excel (Microsoft 365). Statistical significance was assumed at *p* < 0.05.

## Figures and Tables

**Table 1:** Overview of experimental data. Each measurement point includes a fast chlorophyll fluorescence rise (OJIP) curve and a set of 31 different parameters derived from the conventional JIP tests.

	Group	RF-EMF Exposure	Drought Treatment	Number of Plants	Number of Collected Measurement Points
Experiment 1	Group ED	yes	yes	10	785
	Group E	yes	no	10	975
	Group D	no	yes	10	829
	Group Control	no	no	9	739
Experiment 2	Group ED	yes	yes	9	440
	Group E	yes	no	9	476
	Group D	no	yes	9	427
	Group Control	no	no	9	501
Experiment 3	Group ED	yes	yes	9	481
	Group E	yes	no	9	434
	Group D	no	yes	9	471
	Group Control	no	no	9	499
			Total	111	7057

**Table 2:** The table shows the *p*-values and effect sizes (partial eta-squared) of the effect of RF-EMF exposure (left column), the effect of drought treatment (middle column), and the interaction effect between RF-EMF exposure and drought treatment (right column) on the seven previously drought-related parameters. The partial eta squared (η^2^p) was used as the measure of the effect size, with values of 0.01 indicating a small effect, 0.06 a medium effect, and 0.14 a large effect. Statistically significant results are indicated by *p* < 0.05. N.S = not significant.

Drought Response Indicator	1st Experiment			2nd Experiment			3rd Experiment		
	EMF Effect	Drought	Drought-EMF Interaction	EMF Effect	Drought	Drought-EMF Interaction	EMF Effect	Drought	Drought-EMF Interaction
		Effect			Effect			Effect	
F_V_	N.S	N.S	*p* < 0.05	*p* < 0.05	*p* < 0.05	*p* < 0.05	N.S	*p* < 0.05	*p* < 0.05
	η^2^p = 0.001	η^2^p = 0.0	Inverted effect	η^2^p = 0.004	η^2^p = 0.021	η^2^p = 0.003	η^2^p = 0.0	η^2^p = 0.037	η^2^p = 0.004
			η^2^p = 0.002						
Phi(Po) (F_V_/F_M_)	N.S	*p* < 0.05	*p* < 0.05	N.S	*p* < 0.05	*p* < 0.05	*p* < 0.05	*p* < 0.05	*p* < 0.05
	η^2^p = 0.0	η^2^p = 0.08	η^2^p = 0.007	η^2^p = 0.001	η^2^p = 0.151	η^2^p = 0.014	η^2^p = 0.023	η^2^p = 0.093	η^2^p = 0.009
Dio/RC	*p* < 0.05	*p* < 0.05	N.S	*p* < 0.05	*p* < 0.05	*p* < 0.05	*p* < 0.05	*p* < 0.05	*p* < 0.05
	η^2^p = 0.007	η^2^p = 0.042	η^2^p = 0.0	η^2^p = 0.010	η^2^p = 0.073	η^2^p = 0.007	η^2^p = 0.014	η^2^p = 0.022	η^2^p = 0.003
F_O_/F_M_	N.S	*p* < 0.05	*p* < 0.05	N.S	*p* < 0.05	*p* < 0.05	*p* < 0.05	*p* < 0.05	*p* < 0.05
	η^2^p = 0.0	η^2^p = 0.084	η^2^p = 0.007	η^2^p = 0.002	η^2^p = 0.154	η^2^p = 0.015	η^2^p = 0.024	η^2^p = 0.098	η^2^p = 0.01
F_V_/F_O_	N.S	*p* < 0.05	*p* < 0.05	*p* = 0.057	*p* < 0.05	*p* < 0.05	*p* < 0.05	*p* < 0.05	*p* < 0.05
	η^2^p = 0.0	η^2^p = 0.092	0.007	η^2^p = 0.002	η^2^p = 0.154	η^2^p = 0.016	η^2^p = 0.023	η^2^p = 0.103	η^2^p = 0.012
TR/ABS	N.S	*p* < 0.05	*p *< 0.05	N.S	*p* < 0.05	*p* < 0.05	*p* < 0.05	*p* < 0.05	*p* < 0.05
	η^2^p = 0.0	η^2^p = 0.08	η^2^p = 0.008	0.001	η^2^p = 0.15	η^2^p = 0.015	η^2^p = 0.024	η^2^p = 0.098	η^2^p = 0.009
Phi(Po)/(1 − Phi(Po))	N.S	*p* < 0.05	*p* < 0.05	N.S	*p* < 0.05	*p* < 0.05	*p* < 0.05	*p* < 0.05	*p* < 0.05
	η^2^p = 0.0	η^2^p = 0.088	η^2^p = 0.008	η^2^p = 0.002	η^2^p = 0.151	η^2^p = 0.014	η^2^p = 0.022	η^2^p = 0.096	η^2^p = 0.011

**Table 3:** Summarized results of detection of “anomalies” across three experiments.

	Group	RF-EMF Exposure	Drought Treatment	Number of Plants	Number of Collected Measurement Points	Number of Identified “Anomalies”	Mean Percentage of “Anomalies” Per Plant	Median Percentage of “Anomalies” Per Plant
Experiment 1	Group ED	yes	yes	10	785	37	4.7%	4.3%
	Group E	yes	no	10	975	109	11.2%	8.6%
	Group D	no	yes	10	829	56	6.7%	4.9%
	Group Control	no	no	9	739	11	1.5%	1.2%
Experiment 2	Group ED	yes	yes	9	440	44	10.0%	4.1%
	Group E	yes	no	9	476	47	9.9%	7.8%
	Group D	no	yes	9	427	60	14.1%	11.3%
	Group Control	no	no	9	501	5	1.0%	0.0%
Experiment 3	Group ED	yes	yes	9	481	67	13.9%	10.7%
	Group E	yes	no	9	434	37	8.5%	7.5%
	Group D	no	yes	9	471	73	15.5%	16.4%
	Group Control	no	no	9	499	5	1.0%	0.0%

**Table 4:** Comparison of early plant response to drought treatments in group D (drought treatment) and group ED (exposed to RF-EMFs and subjected to drought treatment).

OJIP Parameters	Averaged Values			Is the Difference Between Group D and ED’s “Anomalies” Statistically Significant? (*p* < 0.05)	If Yes, Which Group Displayed Stronger Responses to Drought Treatment? (Group D/Group ED)	Effect Size (Differences Between Group D and ED’s “Anomalies”)
	Group Control	Group D’s Anomalies	Group ED’s Anomalies			
Experiment 1						
F_I_	429	588	601	No	-	
F_M_	622	831	840	No	-	
F_V_	509	688	693	No	-	
F_O_/F_M_	0.182	0.171	0.174	No	-	
F_V_/F_O_	4.53	4.86	4.79	No	-	
F_V_/F_M_ (ϕP_O_)	0.818	0.829	0.826	No	-	
TR_O_/ABS	0.818	0.829	0.825	Yes	Group D	Small
ϕPo/(1 − ϕP_O_)	4.453	4.890	4.788	No	_	
Experiment 2						
F_I_	419	511	509	No	-	
F_M_	622	770	740	No	-	
F_V_	483	627	594	Yes	Group D	Small
F_O_/F_M_	0.223	0.187	0.196	Yes	Group D	Medium
F_V_/F_O_	3.505	4.386	4.137	Yes	Group D	Medium
F_V_/F_M_ (ϕP_O_)	0.777	0.813	0.804	Yes	Group D	Medium
TR_O_/ABS	0.777	0.814	0.804	Yes	Group D	Medium
ϕPo/(1 − ϕP_O_)	3.507	4.377	4.136	Yes	Group D	Medium
Experiment 3						
F_I_	388	611	548	Yes	Group D	Small
F_M_	615	879	787	Yes	Group D	Medium
F_V_	499	725	639	Yes	Group D	Medium
F_O_/F_M_	0.189	0.175	0.187	Yes	Group D	Medium
F_V_/F_O_	4.319	4.735	4.383	Yes	Group D	Medium
F_V_/F_M_ (ϕP_O_)	0.811	0.824	0.813	Yes	Group D	Medium
TR_O_/ABS	0.811	0.825	0.813	Yes	Group D	Medium
ϕPo/(1 − ϕP_O_)	4.323	4.726	4.393	Yes	Group D	Medium

## Data Availability

OJIP data are available in the Supplementary Materials.

## Supplementary Materials

The following supporting information can be downloaded at: https://www.mdpi.com/article/10.3390/ijms26157038/s1.
