# Supplementary material for: In-Depth Analysis of Chlorophyll Fluorescence Rise Kinetics Reveals Interference Effects of a Radiofrequency Electromagnetic Field (RF-EMF) on Plant Hormetic Responses to Drought Stress
Source: Int J Mol Sci. 2025 Jul 22;26(15):7038. doi: 10.3390/ijms26157038 (PMC12345933; doi:10.3390/ijms26157038)
Supplement: Supplementary file 1 [file ijms-26-07038-s001.zip › ijms-3707247-supplementary-final.7.22.pdf]

Supplementary Material

In-depth analysis of chlorophyll fluorescence rise kinetics reveals interference effects of a radio-frequency electromagnetic field (RF-EMF) on plant hormetic responses to drought stress

JULIAN KELLER, JENS UWE GEIER, NAM TRUNG TRAN

1. Data extraction from chlorophyll fluorescence imaging
2. JIP test and their parameters
3. Weather data
4. Model optimization for anomaly detection
5. All fluorescence kinetic data (in Excel file)

1. Data extraction from chlorophyll fluorescence imaging

Recorded chlorophyll fluorescence kinetic data are stored in .tar files that can be opened and processed with FluorCam 10 software. A good measurement typically gives a sharp image of the plant with background fluorescence (F_O) at the level of 450 + 35 a.U. and peak fluorescence (F_M) between 1000 and 2000 a.U. The processing of the data follows the general instructions of the manufacturer.

We divided the recorded image into 120 squares using an 8 × 15 grid, with each square containing 1681 pixels. To ensure reliable analysis, we applied the following filters:

- Only pixels with peak fluorescence above 800 a.U. (background fluorescence + 10 standard deviations) were considered. This removes weak signals and non-plant areas.
- Squares with fewer than 1200 valid pixels were excluded to avoid using data from blurred regions or areas with minimal plant coverage.

For each remaining square, we averaged the data from all valid pixels to generate a single measurement point.

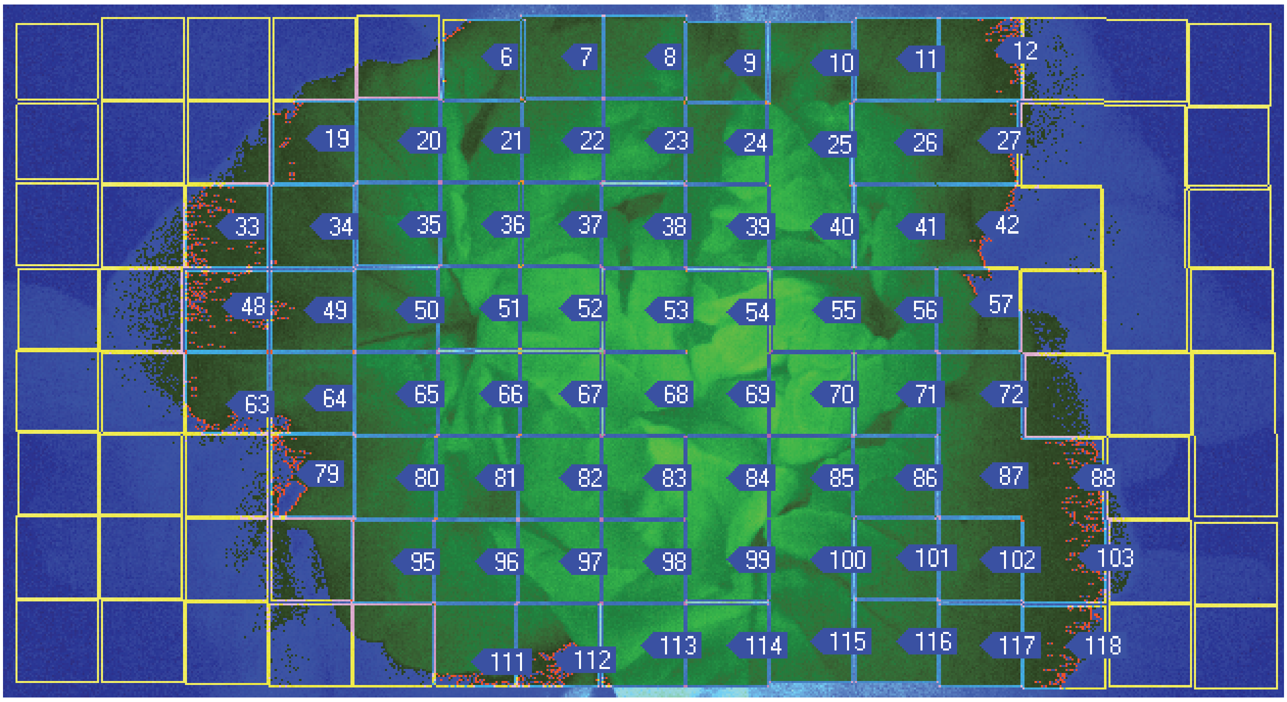

Figure S1: The overlay of an 8x15 grid on a plant image is presented. Each square contains 1681 pixels. Green pixels are considered valid with peak fluorescence above 800 a.U. Conversely, blue pixels have a peak fluorescence below this threshold and are thus not considered in the analysis. Squares marked with numbers indicate those with more than 1200 valid pixels. These squares will be subjected to further analysis.

2. JIP test and their parameters

Table S1: Overview of the JIP test parameters.

Fo	First fluorescence value after the onset of illumination
F300	Fluorescence intensity at 300 ms

Fj	Fluorescence intensity at 2ms (J-Level)
Fi	Fluorescence intensity at 30ms (I-Level)
Fm	Maximal fluorescence intencity
$Fv = F_M – F_0$	Maximum variable Chl fluorescence
Vj	Relative variable fluorescence at 2 ms
Vi	Relative variable fluorescence at 30 ms
Mo	Initial slope of the induction curve
Ss = Vj/Mo	normalized total complementary area corresponding only to the O–J phase<br>(reflecting single-turnover QA reduction events) (paper linden potosynthetika)
Fo/FM	quantum yield (at t = 0) of energy dissipation
Fv/Fo	(Fv/F0) is an indicator of the size and the number of active photosynthetic reaction centers (Linden Paper)<br>Potential photochemical efficiency (Linden Paper)
Phi_Po	maximum quantum yield for primary photochemistry
Phi_ET2o	electron transport quantum yield
Phi_RE1o	quantum yield for reduction of the end electron acceptors at the PSI acceptor side
Psi_ET2o	probability that an electron moves further than QA^-
Psi_RE1o	quantum yield for reduction of the end electron acceptors at the PSI acceptor side
Delta_RE1o	Efficiency/probability with which an electron from QB is transferred until PSI acceptors
ABS/RC	absorption flux per RC
TRo/RC	trapped energy flux per RC
ET2o/RC	Electron transport flux from QA to QB per PSII RC
RE1o/RC	Electron transport flux until PSI acceptors per PSII RC
DIo/RC	dissipate energy flux per RC
TR/ABS	Energy flux ratio between trapping and absorption
ET/TR	energy flux ratio between electron transport and trapping
RE/ET	energy flux ratio between re-emission and electron transport
phi(Po)/(1-phi(Po))	“conformation term“ for primary photochemistry
Psi_ET2o/(1-Psi_ET2o)	“conformation term“ for electron transport beyond QA^-
Delta_RE1o/(1-Delta_RE1o)	“conformation term for the efficiency” of electron transfer to the final PSI acceptors.
PIABS	Performance index for energy conservation from photons<br>absorbed by PSII antenna, to the reduction of QB
PITotal	Performance index for energy conservation from photons absorbed by PSII antenna, until the reduction of PSI acceptors

3. Weather data

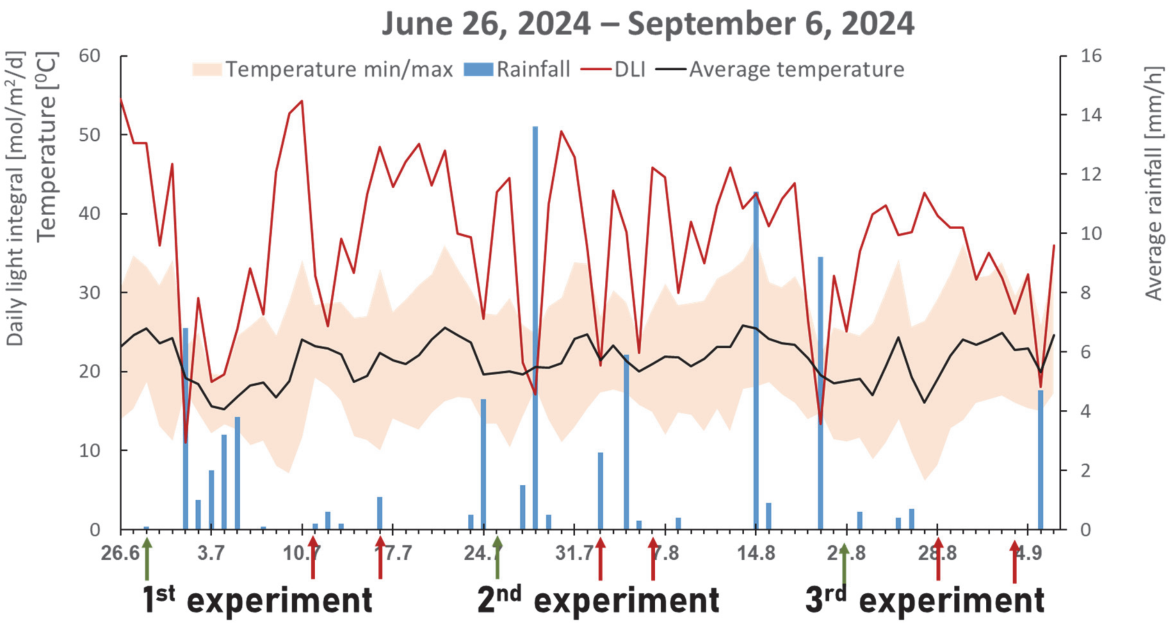

Figure S2: *Weather data throughout the entire experimental period. Green arrows indicate the start of exposure, while red arrows represent the measurement days.*

4. Model optimization for anomaly detection

Anomaly detection was performed on the OJIP kinetic data of the measurement points. Each data point generally comprises 51 attributes, which correspond to the 51 time marks in the OJIP curve. The training set comprises data points from six plants in Group Control. Two datasets were utilized for model validation and assessment. The first validation dataset contains data points from three plants of Group Control that differ from those employed during model training. The second validation dataset comprises data points from all plants of Group D, which are drought-stressed.

The parameters of the model that are subject to optimization are the number of principle components (PCs) and the data preprocessing method. A total of four data preprocessing regimes were evaluated in this study: (1) no preprocessing, (2) column-wise centering, (3) scaling by the standard deviation, and (4) column-wise centering and scaling by the standard deviation. The number of PCs was initially set at 2 and subsequently increased incrementally. The percentage of anomalies typically increases concomitantly.

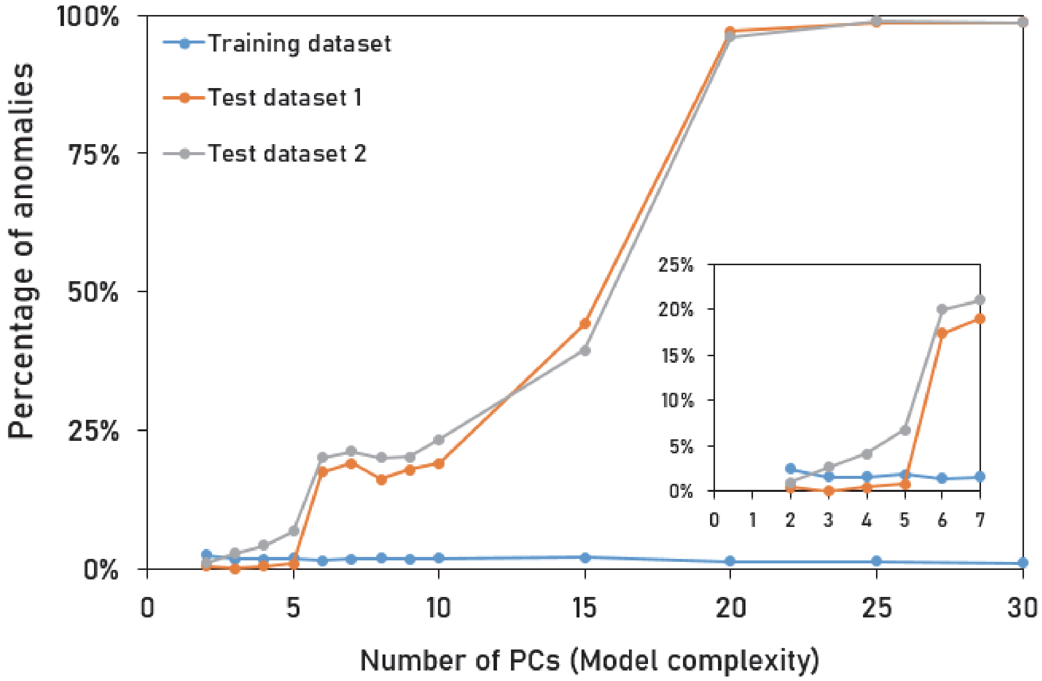

Figure S3: The percentage of identified "anomalies" typically increases concomitantly with the number of Principle Components (PCs) used in model training. The training set comprises data points from six plants in Group Control. The test dataset 1 contains data points from three plants of Group Control that differ from those employed during model training. The test dataset 2 comprises data points from all plants of Group D, which are drought-stressed.

The following criteria were employed for model assessment and optimization:

- With the training dataset, the percentage of anomalies should be approximately 1%.
- With the validation dataset #1, the percentage of anomalies should be less than 200% of that of the training dataset. The optimization of the model was halted once the percentage of anomalies in the validation dataset #1 surpassed 1000% of that observed in the training dataset.
- With the validation dataset #2, the percentage of anomalies should be as high as possible while still meeting the above criteria.

Experiment 1

Table S2: results (number of identified "anomalies") of the model optimisation. Optimum settings are highlighted in yellow.

Data preprocessing	Number of PCs	Training data set (497 instances)	Validation dataset #1 (242 instances)	Validation dataset #3 (829 instances)
No preprocessing	2	9	3	10
	3	13	1	10
	4	8	0	18
	5	8	1	33
	6	9	2	46

	7	7	40	168
	8	7	48	169
	9	9	41	159
	10	8	39	159
Column-wise centering	2	12	1	8
	3	8	0	22
	4	8	1	34
	5	9	2	56
	6	7	42	166
	7	8	46	175
	8	9	39	165
	9	8	43	167
	10	9	46	193
Scaling by the standard deviation	2	15	15	43
	3	12	33	80
	4	11	33	100
	5	12	28	78
Centering and Scaling	2	11	28	62
	3	11	28	64
	4	7	34	133
	5	11	25	89

Experiment 2

Data preprocessing	Number of PCs	Training data set (321 instances)	Validation dataset #1 (180 instances)	Validation dataset #3 (427 instances)
No preprocessing	2	5	0	42
	3	5	4	69
	4	4	1	60
	5	7	8	68
	6	7	9	90
	7	6	11	94
	8	6	14	125
	9	4	33	184
	10	5	62	232
Column-wise centering	2	6	1	49
	3	5	1	52
	4	7	10	59
	5	6	9	87
	6	6	11	91
	7	5	12	121
	8	5	35	190
	9	5	61	236
	10	2	76	278
Scaling by the standard deviation	2	3	52	52
	3	4	56	81
	4	5	57	84
	5	3	58	97
Centering and Scaling	2	3	50	73
	3	3	52	85

	4	4	52	90
	5	6	50	98

Experiment 3

Data preprocessing	Number of PCs	Training data set (333 instances)	Validation dataset #1 (166 instances)	Validation dataset #3 (471 instances)
No preprocessing	2	9	0	51
	3	9	0	56
	4	8	2	57
	5	10	8	92
	6	11	11	83
	7	7	13	150
	8	5	21	180
	9	7	24	192
	10	4	35	238
Column-wise centering	2	10	0	55
	3	11	2	61
	4	10	9	100
	5	11	10	88
	6	9	12	151
	7	5	19	177
	8	5	25	198
	9	5	36	244
	10	6	57	288
Scaling by the standard deviation	2	7	0	32
	3	7	1	33
	4	8	0	50
	5	3	1	66
	6	9	3	98
	7	9	8	141
	8	9	6	139
	9	5	13	155
	10	5	27	189
Centering and Scaling	2	7	1	34
	3	7	1	32
	4	5	0	59
	5	4	1	73
	6	9	2	99
	7	10	7	148
	8	11	5	148
	9	4	14	165
	10	3	29	223
